# Tablet Splitting in Elderly Patients with Dementia: The Case of Quetiapine

**DOI:** 10.3390/pharmaceutics13091523

**Published:** 2021-09-20

**Authors:** Roberta Ganzetti, Serena Logrippo, Matteo Sestili, Alessandro Caraffa, Marco Cespi, Giuseppe Pelliccioni, Paolo Blasi, Giulia Bonacucina

**Affiliations:** 1 Hospital Pharmacy, IRCCS INRCA, Via della Montagnola 81, 60127 Ancona, Italy; roberta.ganzetti@sanita.marche.it; 2 School of Specialization in Hospital Pharmacy, University of Camerino, 62032 Camerino, Italy; serena.logrippo@unicam.it; 3 Hospital Pharmacy, ASUR Marche, Area Vasta 2, 60127 Ancona, Italy; matteo.sestili@sanita.marche.it; 4 Hospital Pharmacy, Hospital S. Maria Della Misericordia, S. Andrea delle Fratte, 06156 Perugia, Italy; alessandro.caraffa@ospedale.perugia.it; 5 School of Pharmacy, University of Camerino, Via Gentile III da Varano, 62032 Camerino, Italy; marco.cespi@unicam.it (M.C.); giulia.bonacucina@unicam.it (G.B.); 6 Department of Neurology, IRCCS INRCA, Via della Montagnola 81, 60127 Ancona, Italy; g.pelliccioni@inrca.it; 7 Department of Pharmacy and Biotechnology, University of Bologna, Via San Donato 19/2, 40127 Bologna, Italy

**Keywords:** behavioral and psychological symptoms of dementia, BPSD, compounding, dysphagia, tablet splitter, kitchen knife, tablet cutter

## Abstract

Quetiapine is an atypical antipsychotic approved for treating schizophrenia, bipolar depression, and mania but is frequently used in an off-label manner to control the behavioral and psychological symptoms of dementia in elderly patients with dementia. Due to the need to personalize doses for elderly patients with dementia, quetiapine tablet manipulation is widespread in hospital settings, long-term care facilities, and patient homes. The aim of this study was to assess the impact of the different splitting techniques on quetiapine fumarate tablets by analysing the obtained sub-divided tablets and to discuss compliance with the European Pharmacopoeia limits on whole and split tablets. Quetiapine fumarate tablets of two dose strengths were taken at random (in a number able to assure a power of 0.8 during statistical comparison) and were split with a kitchen knife or tablet cutter. The weight and the drug content were determined for each half tablet. The obtained data were compared to the European Pharmacopoeia limits. The differences between the different splitting techniques were statistically tested. Data showed that split tablets, independently of the dose strength and the technique employed, were not compliant with the European Pharmacopoeia specifications for both entire and subdivided tablets in terms of weight and content uniformity. Thus, such a common practice could have potential effects on treatment efficacy and toxicity, especially when also considering the fragility of the elderly target population in which polypharmacotherapy is very common. These results indicate a compelling need for flexible quetiapine formulations that can assure more accurate dose personalization.

## 1. Introduction

Patients with Alzheimer’s disease, vascular dementia, Parkinson’s dementia, fronto-temporal dementia, and Lewy body dementia can develop changes in behaviour and personality. The frequency and nature of these symptoms might vary over the course of the disease [1]. In the majority of patients, lack of insight occurs even in the early stages of the disease and represents an important predictive factor for the occurrence of neuropsychiatric symptoms, including apathy, agitation, irritability, psychosis, or behavioral symptoms in general [2].

Atypical antipsychotic medications are the only class of drugs showing consistent benefit in controlling the behavioral and psychological symptoms of dementia (BPSD) for short-term treatment [3,4]. BPSD include agitation, verbal aggression, sleep disturbances, hallucinations, persecutory delirium, psychosis, and mood disorders and are associated with high levels of distress in both patients and caregivers [5]. BSD are a significant clinical target for intervention [6], even if the use of antipsychotics must be balanced against their serious adverse effect profile [7].

The market mainly offers solid oral dosage forms (SODFs) of psychotropic drugs that do not cover all the patient needs, so tablet manipulation (splitting and/or crushing) is often required and has become a common practice [8]. On the one hand, tablet manipulation allows dose flexibility and facilitates swallowing, which is often compromised in advanced phases of dementia, but on the other hand, it can alter the dosage form performance [9,10,11]. Tablet splitting may be particularly difficult if the tablets do not have break marks, and inappropriate manipulation can lead to the administration of an imprecise dose [12,13].

Since the global prevalence of dementia (5–7% of world population in 2010) is expected to increase due to the aging of the population, the clinical needs for suitable dosage forms will increase as well [14,15].

In Italy, neurologists from the Alzheimer Evaluation Centers can prescribe atypical antipsychotics to patients diagnosed with dementia for the treatment of BPSD, using an ad hoc “treatment plan” to be updated every 2 months. The treatment plan contains the diagnosis of BPSD, personal data, patient primary disease as well as the patient’s prescribed therapy and dosage regimen. Atypical antipsychotics are dispensed by hospital pharmacies after the submission of the treatment plan to the pharmacist. Quetiapine (2-(2-(4-Dibenzo(b,f)(1,4)thiazepine-11-yl-1-piperazinyl)ethoxy)ethanol) is an atypical antipsychotic with higher affinity for the serotonin 5-HT2A receptor than for the D2 receptor in the brain and with minimal extrapyramidal side effects [16]. While it has been approved by the European Medicine Agency for treating schizophrenia, bipolar depression, and mania [17,18,19], due to its favourable properties, it is very frequently used in an off-label manner to control BPSD in elderly patients with dementia.

Taking into account the widespread prescription of quetiapine fumarate tablets for BPSD treatment in elderly patients with dementia and the specific need for dose adjustment as a function of clinical response, quetiapine tablet manipulation is widespread in hospital settings, long-term care facilities, and patient homes. Thus, the aim of this study was to assess the impact of the different splitting techniques on quetiapine fumarate tablets by analysing the obtained half tablets. In particular, the quality of the split tablets, intended as the uniformity of dosage units (content uniformity and weight variation), was assessed, and the compliance with the limits imposed by the European Pharmacopoeia in the case of whole and split tablets was discussed.

## 2. Materials and Methods

### 2.1. Data Collection on Splitting Techniques

Under the auspices of the Report-AGE project, during the summer of 2016, hospital pharmacists at the Italian National Research Centers on Aging (INRCA) collected data from patients with a BPSD diagnosis and who were treated with quetiapine fumarate (*N* = 155). From patient treatment plans, they identified a sub-group of subjects (*n* = 52) who had been prescribed a dose that required 25 and/or 100 mg tablets to be split. The characteristics of the sample population are reported in Table 1.

During drug dispensing, the hospital pharmacist collected information about the technique adopted by patients or family caregivers for tablet splitting.

### 2.2. Effect of Splitting Technique on Tablets Weight and Dose

#### 2.2.1. Materials

Standard quetiapine fumarate was purchased from Sigma-Aldrich (Darmstadt, Germany; batch number 2501103), while 25 and 100 mg quetiapine fumarate tablets (Quetiapina Teva) were from Teva Pharmaceutical Industries Ltd. (Teva Italia S.r.l. Milan, Italy).

The Quetiapina Teva 25 mg tablets were immediate-release tablets without a score line with a mean weight of 62.9 mg (SD ± 0.8) and a size of ~ 5.1 mm in diameter and ~2.8 mm in thickness. The Quetiapina Teva 100 mg tablets were immediate-release tablets without a score line with a mean weight of 254.0 mg (SD ± 2.3) and a size of ~8.7 mm in diameter and ~4.4 mm in thickness.

Analytical grade potassium dihydrogen phosphate and hydrochloric acid 37% were purchased from AppliChem GmbH (Darmstadt, Germany) and Sigma-Aldrich (Seelze, Germany), respectively.

#### 2.2.2. Sample Size

The actual mean weight and standard deviation of the 25 and 100 mg quetiapine fumarate tablets were determined using an analytical balance (Gibertini E42S, sensitivity 0.1 mg; Gibertini Elettronica, Milan, Italy).

To perform weight and drug content analysis of variance, sample size was determined for the whole and split tablets. Power analysis was conducted by setting the effect size at 5%, a standard deviation equal to the double of that was preliminarily measured, and a power of 0.8 was achieved. In the case of drug content power analysis, the standard deviation was derived from the coefficient of variation of tablet weight by hypothesizing a homogenous distribution of the active pharmaceutical ingredients in the tablet mass.

The sample size that was obtained was 5 tablets for the weight and 6 tablets for the drug content. Out of caution, the sample size was increased to 12 tablets for each group, obtaining a theoretical power around 0.8.

#### 2.2.3. Sample Preparation and Data Collection

The actual weight of twelve tablets for each dose strength was determined using an analytical balance (Gibertini E42S, sensitivity 0.1 mg; Gibertini Elettronica, Milan, Italy), and the calculated half weight of each tablet was used as a control.

Twelve tablets of quetiapine fumarate were taken at random, split with a kitchen knife or tablet cutter (Ultra pill splitter, Apex^®^—Carex^®^ Health Brands, a wholly-owned subsidiary of Compass Health Brands, Middleburg Heights, OH, USA), and positioned inside a specific grid. For each tablet, only one half (right or left alternatively, Figure S1) was collected and used for the analysis. The same procedure was applied to the 100 mg and 25 mg quetiapine tablets. The weight of each half tablet obtained after splitting was determined using the analytical balance.

The drug content for each half tablet or whole tablet was determined by UV-Vis analysis (Shimadzu UV-1800 spectrophotometer, equipped with the UV-Probe 2.43 software, Shimadzu, Kyoto, Japan) following the procedure reported by Pucci et al. [20]. Each dose was dissolved in a suitable amount of phosphate buffer pH 2.5 to obtain a nominal concentration of 50 µg/mL, and the absorbance was measured at 246 nm. The drug content was determined through a calibration curve obtained by analysing standard solutions of quetiapine fumarate in phosphate buffer with a pH 2.5 in the concentration range 8.13–65.00 µg/mL (*r*^2^ > 0.999). All analyses were performed in triplicate. The calculated half content of the whole quetiapine tablets was used as a control.

#### 2.2.4. Statistical Analysis

Descriptive statistics for the two measured parameters (weight and drug content) is reported for all the groups.

Differences between the two different splitting techniques were evaluated using the *t*-test, setting the minimum level of significance at 5%. The normality of the data distributions was evaluated with the Shapiro–Wilk normality test, setting the α level of 0.05, while the homoscedasticity (equality of variance) of the two compared groups was evaluated using the F-test at a 95% confidence interval (α = 0.05).

Pearson’s correlation test was performed to determine any correlation between the weight and the drug content for each group. The correlation coefficients and the associated probability values were calculated, and a minimum level of significance of *p* < 0.05 was used.

## 3. Results

### 3.1. Data about Splitting Techniques

During the summer of 2016, the hospital pharmacy identified 155 patients with prescriptions for 100 and 25 mg quetiapine fumarate tablets. Of these patients, 34% (*n* = 52) had prescriptions requiring tablet splitting. Most of these patients (75%) were over 80 years of age, and Alzheimer’s disease was the main cause of dementia (≈69%) (Table 1).

The use of 25 mg split tablets occurred in 16 patients, while the use of 100 mg split tablets occurred in 36 patients (for 10 of whom the caregiver split and crushed the tablets).

**Figure 1:**
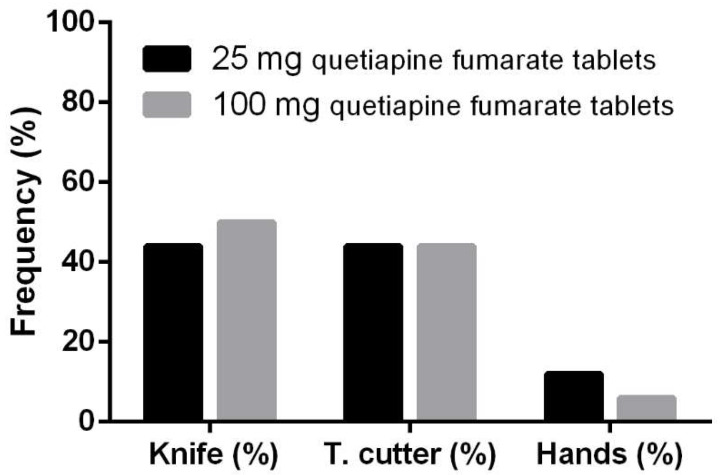
Frequency of tablet splitting techniques reported by patients and/or family caregivers.

In both cases, kitchen knives and tablet cutters were the two most common tools for tablet splitting, with a frequency higher than 88% (Figure 1).

For 23% of all patients (*N* = 155), the tablets were crumbled and dispersed in liquid or in a semisolid vehicle to facilitate swallowing, and, out of these, for 30% (11 patients), this operation was conducted using half tablets.

### 3.2. Effect of Splitting Technique on Tablet Weight and Dose

**Figure 2:**
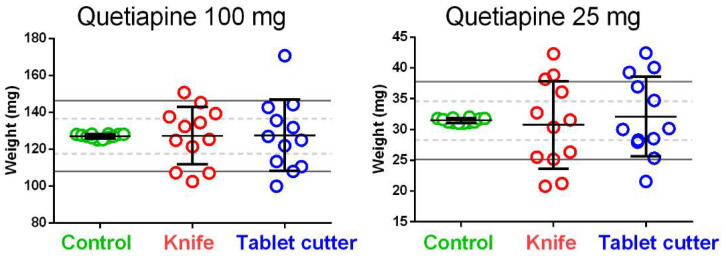
Effect of tablet splitting on weight for 100 mg and 25 mg quetiapine tablets. The control groups are represented by the half of the whole tablet weights. The central black lines represent the mean values, while the whiskers represent the standard deviations. The solid grey lines and the dashed grey lines in the background represent the European Pharmacopoeia limits for the uniformity of mass of single-dose preparations (quetiapine 100 mg: solid grey lines ± 15% and dash grey lines ± 7.5%; quetiapine 25 mg: solid grey lines ± 20% and dash grey lines ± 10%) For simplicity, the European Pharmacopoeia limits showed in the plots have been calculated on the mean weight values of the control groups.

The effect of tablet splitting on the weight is reported in Figure 2. Independently of the quetiapine dose, split tablets showed very scattered weights compared to the control groups. The coefficient of variation for the weight of the control tablets was around 18 and 15 times lower than that obtained for the split tablets for quetiapine 100 mg and 25 mg, respectively (Table 2).

**Figure 3:**
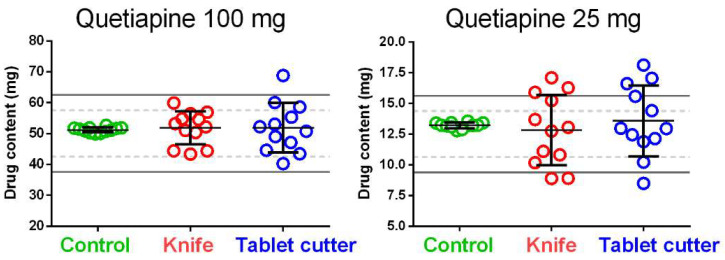
Effect of tablet splitting on drug content for 100 mg and 25 mg quetiapine tablets. The control groups are represented by the half drug content of the whole tablet contents. The central black lines represent the mean values, while the whiskers the standard deviations. The solid grey lines and the dashed grey lines in the background represent the European Pharmacopoeia limits for the uniformity of content of single-dose preparations: solid grey lines ± 25%, and dash grey lines ± 15%. For simplicity, the European Pharmacopoeia limits showed in the plots have been calculated on the mean drug content values of the control groups.

Similar results were obtained for the drug content (Figure 3). In the group of half-tablets (split by knife or cutter), where the masses were strongly scattered, the drug amount was highly variable: the coefficient of variation for the drug content of the control tablets was around 13 and 8 times lower than those obtained for the split tablets for quetiapine 25 mg and 100 mg, respectively (Table 2).

**Figure 4:**
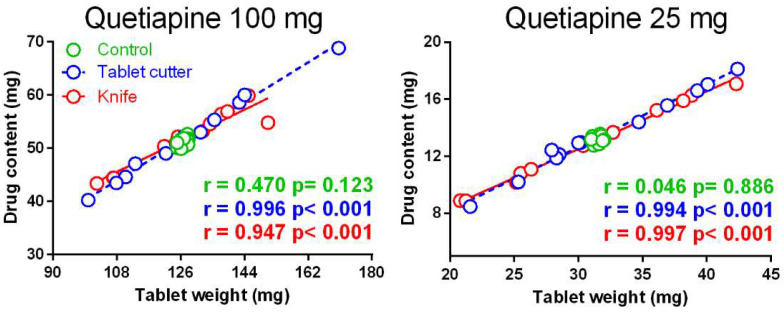
Pearson correlation analysis between weight and drug content for all the three the groups of half tablets: control (the values refer to the half weight or half content of the whole tablets), tablet cutter, and knife.

The relationship between weight and drug content was analysed by Pearson correlation analysis (Figure 4). In the groups of half tablets (split by knife or cutter) a strong positive (*r* ≥ 0.947), highly significant (*p* < 0.001) correlation was found between weight and drug content, indicating a good tablet content uniformity. It can be stated that a variation of the tablet mass determines a change in the drug content. On the other hand, in the control groups, the correlation was weak and never statistically significant, as expected from the scarce variability of the tablet weights (Table 2). Consequently, any correlation is due only to chance.

The comparison among knife and tablet cutter groups was performed by *t*-test. The results of the statistical analysis highlighted the absence of any significant differences between the weight of the half tablets obtained with the knife and the tablet cutter for the two types of tablets (Table 2).

## 4. Discussion

Quetiapine prescriptions for the control of BPSD that require tablet splitting are common among elderly patients. In fact, the highest frequency of prescribed doses achievable through tablet splitting is found for psychotropic drugs in elderly patients [21,22]. Of note, in 2011, quetiapine was the antipsychotic drug most often prescribed as a half tablet (tablet without score lines) at the University Hospital in Basel [23]. Nevertheless, limited information is available on techniques exploited by patients or family caregivers to split tablets. Data show that tablet splitters are rarely used by patients [24,25]. When patients experience difficulties with hand or teeth splitting, knives or other sharp objects are preferred to tablet splitters [26]. Instead, in the present study, these observations do not apply since almost half of the patients use a tablet splitter, and only a marginal fraction split tablets by hand.

The analysis of the weight and drug content of the split tablets (split with a tablet cutter or kitchen knife) highlighted the high dispersion of the measured values around the mean.

The tablets that were investigated were immediate-release tablets without a score line for which tablet subdivision to deliver fractional doses is not contemplated. In addition, being tablet fractional doses, the European Pharmacopoeia tests for single-dose preparations do not apply. In the absence of specific tests, the quality of sub-divided tablets has been discussed by comparing our results with the European Pharmacopoeia limits indicated on the uniformity of mass of single-dose preparations, the uniformity of the content of single-dose preparations, and the subdivision of tablets [27,28,29].

None of these groups were compliant with the European Pharmacopoeia specifications (2.9.5. uniformity of mass of single-dose preparations). In the case of quetiapine 100 mg, no more than two of the individual masses may deviate from the average mass by more than 7.5% and none by more than 15%, while for quetiapine 25 mg, no more than two of the individual masses may deviate from the average mass by more than 10% and none by more than 20% [27].

In addition, they were not compliant with European Pharmacopeia standards for scored-tablet subdivision of tablets, which state that no more than one individual mass (on 30 half tablets) can be outside the limit of 85–115% of the average mass [29].

Interestingly, such results were obtained by analysing only 12 tablets against the 20 or 30 tablets specified in the European Pharmacopeia, indicating that split tablets are very far from the quality requirements mandatory for industrial medicinal products. It should be borne in mind that these results were obtained with experienced laboratory personnel operating in ideal conditions. Greater variations in quetiapine content should be expected in real-life situations since patients and/or caregivers perform tablet splitting less precisely and in a less-than suitable environment.

Concerns about weight and drug content accuracy when tablets are split were first raised between the 1980s and 1990s [30,31] and more have been extensively studied in the last 15 years. Published results vary considerably, which is probably due to the specific features of the analysed tablets. In fact, it has been reported that the presence of scoring lines [12,32,33,34], tablet shape and hardness [32,34,35], tablet size [33,35], splitting techniques [13,33,36], and patient training [37] affect the accuracy of the splitting procedure. In the specific case of quetiapine fumarate tablets, the marked weight variation could be correlated to the absence of score lines and the small tablet size. The latter point appears particularly significant: the 25 mg tablets, which are about 5.1 mm in diameter and 2.8 mm thick, show almost twice the variability (on the CV values) as the 100 mg tablets, which are about 8.7 mm in diameter and 4.4 mm thick. This is in agreement with previous findings that tablets with a diameter smaller than 7/8 mm are difficult to handle and tend to break [38]. Contrary to published results, we observed that the use of a tablet cutter does not assure higher accuracy than the use of a kitchen knife [36]. Although not investigated in the present work, the amount of weight loss during the tablet splitting procedure should also be considered since it would further reduce the accuracy of the administered dose.

Tablet splitting is a quite common practice, but its effect on medicinal product quality have been largely overlooked [36]. Here, we demonstrate that quetiapine split tablets are not compliant with the European Pharmacopeia quality requirements and thus may affect treatment efficacy and toxicity.

Several new atypical antipsychotic drugs, including quetiapine, risperidone, olanzapine, and ziprasidone, have become available for the short-term treatment of BPSD. Long-term use of antipsychotics in individuals with dementia is frequent notwithstanding guidelines that recommend time-limited use in treatment [39]. Available data also showed that antipsychotics are often used in patients for sustained periods (>6 months), with limited monitoring of their effects [40]. Atypical antipsychotics are considered to have a favourable adverse effect profile compared to traditional antipsychotics although, in rare cases, therapeutic and supratherapeutic doses have led to the death of patients [41]. Even if quetiapine is reportedly well tolerated in therapeutic doses, overdoses are characterized by hypotension, sinus tachycardia, and somnolence due to central nervous system depression [42]. These adverse effects could be increased by the co-ingestion of other drugs with similar metabolic pathways, in particular those that inhibit cytochrome P450 isoenzyme CYP3A4 and CYP3A5 [43]. On the other hand, underdosages may compromise the efficacy of quetiapine.

## 5. Conclusions

Patients treated for BSPD with quetiapine are often obliged to divide tablets in order to achieve the prescribed dose. Probably due to the small size of the tablets, most of the patients interviewed divide the tablets with a kitchen knife or tablet cutter. In this study, we have found that the splitting of quetiapine tablets, independently of the dose strength and the tool employed, compromises the dose accuracy, in that the variation of weight and drug content uniformity falls outside of the limits established by the European Pharmacopoeia.

We therefore consider that in elderly patients with dementia, a population which tends to be in polypharmacotherapy, it is of prime importance to avoid potential side effects by ensuring correct dosing. Formulations such as oral suspensions would allow more precise dose personalization, but to date, they are an unmet need [44].

## Figures and Tables

**Table 1:** Characteristics of the sample population (*n* = 52) diagnosed with BPSD, for whom tablet splitting was needed to adjust the prescribed dose.

	Number	Percentage
All	52	100
Female	30	57.7
Male	22	42.3
Mean Age ± SD (years)	85.5 ± 7.2	-
65–80	13	25.0
>80	39	75.0
Diagnosis Of Dementia		
Alzheimer’s	36	69.2
Vascular	8	15.4
Parkinson’s	4	7.7
Front-Temporal	3	5.8
Mixed	1	1.9

**Table 2:** Descriptive statistics of all groups analysed, F-test and *t*-test results, and results of Pearson correlation analysis.

		Tablets, Quetiapine 100 mg			Tablets, Quetiapine 25 mg		
		Control	Knife	T. Cutter	Control	Knife	T. Cutter
**Weight comparison**	Mean (mg) ^1^	127.0	127.3	127.5	31.5	30.8	32.1
	SD ^2^	1.2	15.6	19.3	0.4	7.1	6.5
	CV (%) ^3^	0.9	12.2	15.2	1.2	23.1	20.2
	SEM ^4^	0.3	4.5	5.6	0.1	2.1	1.9
	Normal distribution ^5^	Yes	Yes	Yes	Yes	Yes	Yes
	Homoscedasticity ^6^	/	Yes		/	Yes	
	*t*-test	/	*p*-value = 0.977		/	*p*-value = 0.633	
**Drug content comparison**	Mean (mg) ^1^	51.2	51.8	52.0	13.2	12.8	13.6
	SD ^2^	0.8	5.4	8.0	0.2	2.9	2.9
	CV (%) ^3^	1.6	10.3	15.4	1.7	22.5	21.2
	SEM ^4^	0.2	1.5	2.3	0.1	0.8	0.8
	Normal distribution ^5^	Yes	Yes	Yes	Yes	Yes	Yes
	Homoscedasticity ^6^	/	Yes		/	Yes	
	*t*-test	/	*p*-value = 0.962		/	*p*-value = 0.530	
**Pearson correlation**	Correlation coefficient (r)	0.470	0.947	0.996	0.046	0.997	0.994
	*p*-value	0.123	<0.001	<0.001	0.886	<0.001	<0.001

^1^ Mean weight of half tablets (for the control group, the mean value refers to the half weights of the whole tablets). ^2^ Standard deviation. ^3^ Coefficient of variation (relative standard deviation RSD %). ^4^ Standard error of mean. ^5^ The normality of distributions was tested using the Shapiro–Wilk normality test with an α level of 0.05. ^6^ The homoscedasticity (equality of variance) of the two distributions compared to the t-test was evaluated using the F test with α level of 0.05.

## Supplementary Materials

The following are available online at https://www.mdpi.com/article/10.3390/pharmaceutics13091523/s1, Figure S1: Sample picture of twelve split tablets (25 mg quetiapine fumarate) positioned inside a specific grid to select the samples for analysis.
