# Supplementary material for: Tablet Splitting in Elderly Patients with Dementia: The Case of Quetiapine
Source: Pharmaceutics. 2021 Sep 20;13(9):1523. doi: 10.3390/pharmaceutics13091523 (PMC8469994; doi:10.3390/pharmaceutics13091523)
Supplement: Supplementary file 1 [file pharmaceutics-13-01523-s001.zip › pharmaceutics-1323136-supplementary.pdf]

pharmaceutics

Supplementary Materials: Tablet Splitting in Elderly Patients with Dementia: The Case of Quetiapine

Roberta Ganzetti, Serena Logrippo, Matteo Sestili, Alessandro Caraffa, Marco Cespi, Giuseppe Pelliccioni, Paolo Blasi and Giulia Bonacucina

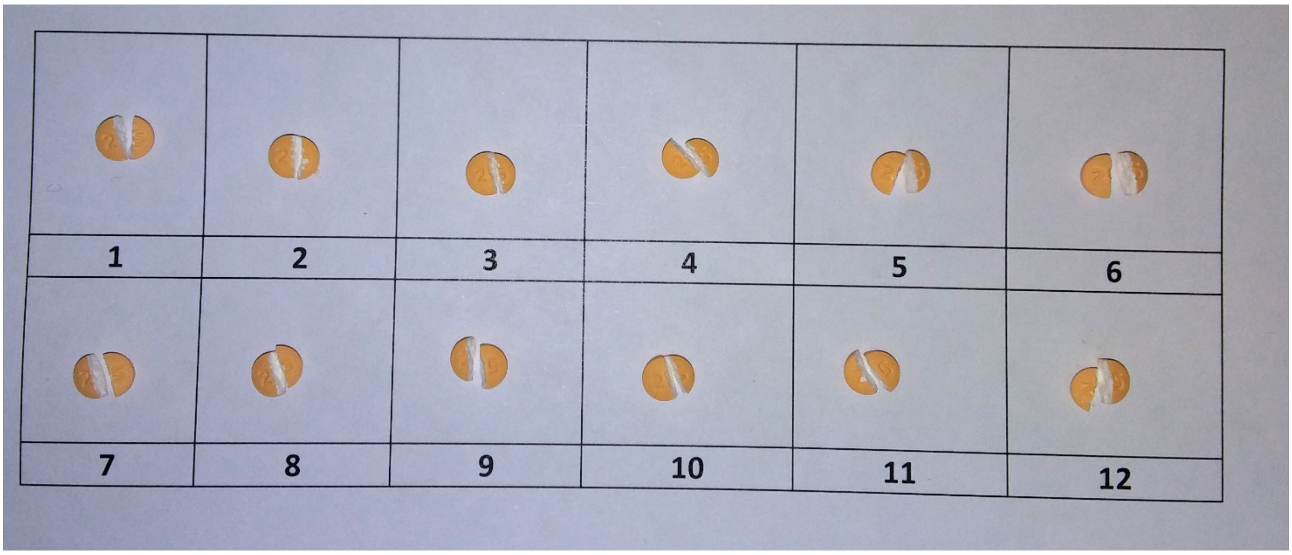

Figure S1. Sample picture of twelve split tablets (25 mg quetiapine fumarate) positioned inside a specific grid to select the samples for analysis.
